# Menstrual migraine is caused by estrogen withdrawal: revisiting the evidence

**DOI:** 10.1186/s10194-023-01664-4

**Published:** 2023-09-21

**Authors:** Bianca Raffaelli, Thien Phu Do, Basit Ali Chaudhry, Messoud Ashina, Faisal Mohammad Amin, Håkan Ashina

**Affiliations:** 1 Department of Neurology, Danish Headache Center, Copenhagen University Hospital - Rigshospitalet, Copenhagen, Denmark; 2 Department of Neurology, Charité Universitätsmedizin Berlin, Berlin, Germany; 3 Clinician Scientist Program, Berlin Institute of Health at Charité (BIH), Berlin, Germany; 4 Department of Clinical Medicine, University of Copenhagen, Copenhagen, Denmark; 5 Danish Knowledge Center On Headache Disorders, Glostrup, Denmark; 6 Department of Brain and Spinal Cord Injury, Copenhagen University Hospital - Rigshospitalet, Copenhagen, Denmark; 7 Department of Anesthesia, Critical Care and Pain Medicine, Beth Israel Deaconess Medical Center, Harvard Medical School, Boston, MA USA; 8 Harvard Medical School, Boston, MA USA

**Keywords:** Menstrual migraine, Estrogen, Estrogen withdrawal hypothesis, Critical appraisal

## Abstract

**Objective:**

To explore and critically appraise the evidence supporting the role of estrogen withdrawal in menstrual migraine.

**Main body:**

Menstrual migraine, impacting about 6% of reproductive-age women, manifests as migraine attacks closely related to the menstrual cycle. The estrogen withdrawal hypothesis posits that the premenstrual drop in estrogen levels serves as a trigger of migraine attacks. Despite its wide acceptance, the current body of evidence supporting this hypothesis remains limited, warranting further validation. Estrogen is believed to exert a modulatory effect on pain, particularly within the trigeminovascular system – the anatomic and physiologic substrate of migraine pathogenesis. Nevertheless, existing studies are limited by methodologic inconsistencies, small sample sizes, and variable case definitions, precluding definitive conclusions. To improve our understanding of menstrual migraine, future research should concentrate on untangling the intricate interplay between estrogen, the trigeminovascular system, and migraine itself. This necessitates the use of robust methods, larger sample sizes, and standardized case definitions to surmount the limitations encountered in previous investigations.

**Conclusion:**

Further research is thus needed to ascertain the involvement of estrogen withdrawal in menstrual migraine and advance the development of effective management strategies to address unmet treatment needs.

## Background

Migraine is a disabling neurologic disease that affects more than one billion people worldwide, predominantly females [1]. Characteristic features include recurrent attacks of headache and accompanying symptoms such as photophobia, phonophobia, and nausea or vomiting [2]. Some affected people also experience transient neurologic disturbances, referred to as migraine aura, which tend to precede or accompany the headache [2].

An interesting clinical observation is the significant number of women of reproductive age who report migraine attacks, with and without aura, in relation to their menstruation [3–5]. This observation gave rise to the term ‘menstrual migraine’ and provided a foundation for understanding the disorder [6]. There are several hypotheses proposed to explain the etiology of menstrual migraine, with the estrogen withdrawal hypothesis being the most widely accepted. This hypothesis was first introduced by Brian W. Sommerville in 1972 [7], based on his experimental studies suggesting that the precipitous drop in estrogen shortly before menstruation increases the risk of developing a migraine attack [7–9]. However, conflicting results have since emerged, and the underlying mechanisms of menstrual migraine are still not entirely clear.

In this review, we undertake a critical appraisal of the estrogen withdrawal hypothesis, a long-standing concept to understanding menstrual migraine. Mindful of the rapidly evolving field of sex differences in migraine, we confine our focus to this particular hypothesis, which has shaped scientific discourse and exploration of menstrual migraine for more than five decades. Through a synthesis of evidence both supporting and contesting the estrogen withdrawal hypothesis, we elucidate existing gaps in understanding, aiming to stimulate further investigation into the exact disease mechanisms at play in menstrual migraine.

## Terminology

### Menstrual cycle

**Fig. 1:**
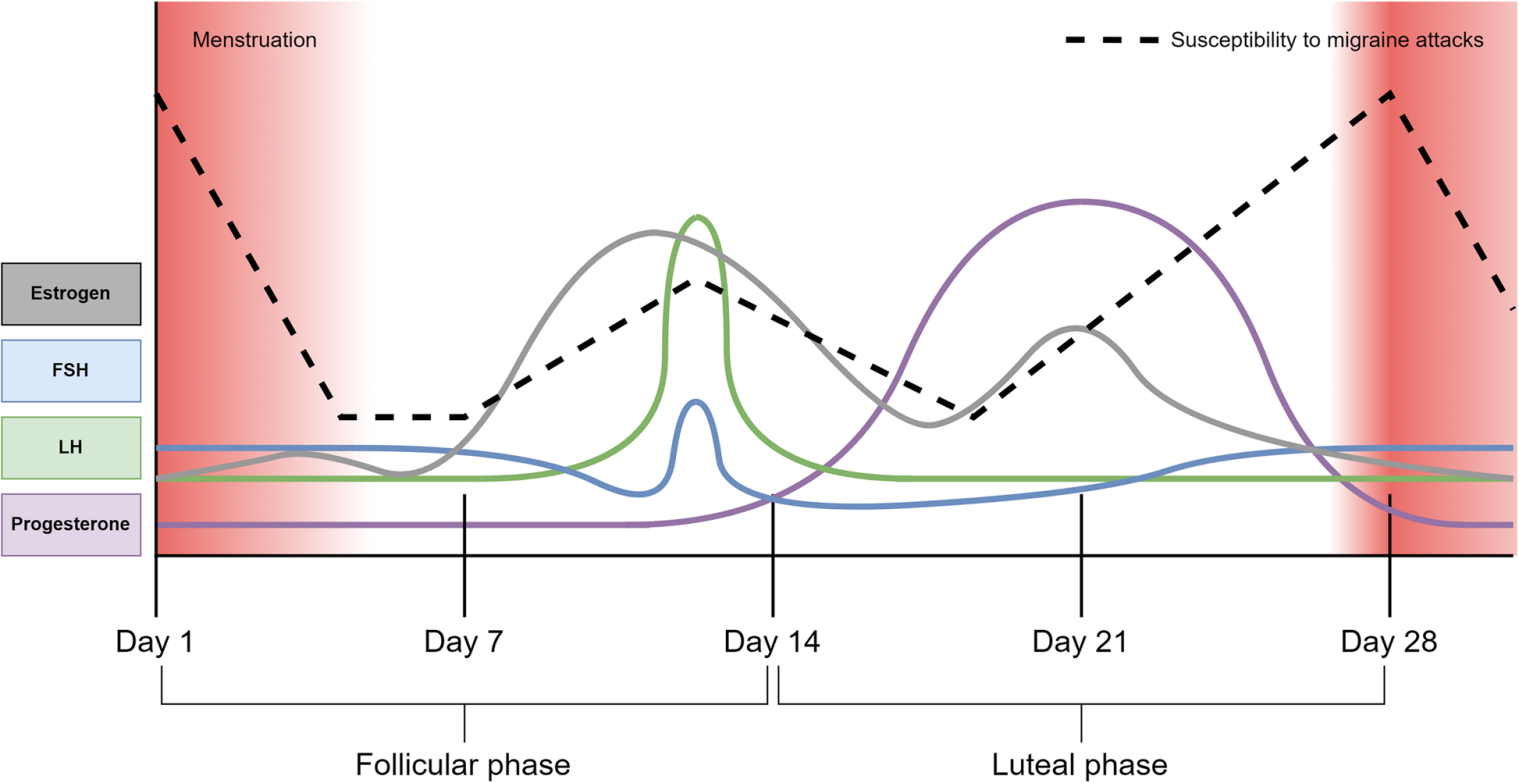
Hormonal changes and incidence of migraine during the menstrual cycle

The human menstrual cycle has an average length of 28 days and is divided into a follicular phase and luteal phase, lasting approximately 14 days each [10, 11]. Each phase is furthermore stratified into an early-, mid-, and late-phase. The follicular phase commences on the first day of menstrual bleeding (i.e., day 1 of the menstrual cycle) and ends with ovulation around day 14 [10, 11]. The luteal phase then begins the day after ovulation and ends just before menstrual bleeding [10, 11]. Fluctuating levels of sex hormones regulate the menstrual cycle and include estrogen and progesterone [10, 11]. The concentration of estrogen is at its lowest during menstruation and then rises steadily throughout the follicular phase to peak the day before ovulation [10, 11]. A decline in estrogen levels then follows before a secondary smaller rise occurs during the mid-luteal phase. This concludes with a precipitous drop in estrogen levels, resulting in the onset of menstruation [10, 11]. An overview of the menstrual cycle and the related fluctuating levels of sex hormones are shown in Fig. 1.

### Menstrual migraine

The diagnosis of migraine is established based on clinical criteria provided by the International Classification of Headache Disorders (ICHD) [12]. The 1st edition of the ICHD from 1988 did not include distinct diagnostic criteria for menstrual migraine but did provide some commentary [13]. The classification committee suggested that menstrual migraine can be used to describe a condition in which at least 90% of migraine attacks occur on day 1 ± 2 (i.e., days − 2 to + 3) of menstruation [13]. This definition was later revised in the 2nd edition of the ICHD from 2004 [14], in which appendix criteria were outlined for two types of menstrual migraine without aura: “pure menstrual migraine” and “menstrually-related migraine”. The same appendix criteria have been maintained in more recent editions of the ICHD, with the addition of criteria for both types of menstrual migraine in patients with aura symptoms [12]. Table 1 shows the appendix criteria for pure menstrual migraine and menstrually-related migraine, as described in the 3rd edition of the ICHD from 2018 [12]. Of note, the ICHD classifies menstruation as endometrial bleeding due to either the normal menstrual cycle or withdrawal bleeding during hormone-free intervals in regimens with hormonal contraception [12].

**Table 1:** The diagnostic criteria for menstrual migraine according to the International Classification of Headache Disorders 3 (ICHD-3) [12]

**Pure Menstrual Migraine**	
A	Attacks, in a menstruating woman, fulfilling criteria for migraine without/with aura and criterion B below
B	Occurring exclusively on day 1 ± 2 (i.e., days − 2 to + 3) of menstruation1 in at least two out of three menstrual cycles and at no other times of the cycle
**Menstrually-Related Migraine**	
A	Attacks, in a menstruating woman, fulfilling criteria for migraine without/with aura and criterion B below
B	Occurring on day 1 ± 2 (i.e., days − 2 to + 3) of menstruation in at least two out of three menstrual cycles, and additionally at other times of the cycle.

## Epidemiology

The 1-year prevalence of migraine is estimated to be 15% in the general population, with a 3:1 female to male ratio [15]. The incidence rises steeply during adolescence and early adulthood, with about 50% of affected people reporting onset of migraine before the age of 25 years [16]. A familial aggregation of migraine is now well-established, and genetic factors play a major role in migraine etiology [2].

The prevalence of menstrual migraine is not well understood, primarily due to the scarcity of data and limitations in population-based studies that differ in case definitions and assessment methods used [17–21]. A Dutch study recruited 1181 women aged 13–55 years from the general population and evaluated for the presence of menstrual migraine based on a questionnaire [18]. The study found that 3% of women reported migraine attacks between day − 2 and day + 2 of the menstrual cycle, with 0.9% of women reporting attacks exclusively during this time. A Norwegian population-based study screened 3,514 women aged 30–34 years for possible menstrual migraine using a questionnaire and invited potential cases for a semi-structured interview to confirm adherence with the ICHD-3β appendix criteria [17]. The results showed that among women aged 30–34 years in the general population, 0.8% have pure menstrual migraine without aura, and 0.1% have pure menstrual migraine with aura. The corresponding figures were 5.3% for menstrually-related migraine without aura and 0.6% for menstrually-related migraine with aura. However, the Dutch and Norwegian population-based data must be interpreted with caution [17, 18]. A recent clinic-based study found that the accuracy of self-reported menstrual migraine is poor, compared with a diary-based diagnosis [22]. Future estimates on the prevalence of menstrual migraine should be based on prospectively collected data from headache diaries with daily entries and time stamps over at least three months.

## Clinical presentation

Menstrual migraine can present with distinct clinical features that distinguish it from attacks outside the perimenstrual period. Diary-based studies have found that perimenstrual attacks are often more disabling and can persist up to 35% longer than those unrelated to the menstrual cycle [23–27]. Furthermore, some evidence suggests that perimenstrual attacks are associated with more severe pain and accentuated photophobia and phonophobia [23]. The therapeutic response to triptans also seems to differ between perimenstrual attacks and those outside the perimenstrual period, with the former having a higher recurrence rate of attacks after treatment [23]. Taken together, these observations suggest the need for further inquiries into the unique clinical features of perimenstrual migraine attacks.

## Migraine pathogenesis

The trigeminovascular system is the anatomic and physiologic framework of migraine [28]. Nociceptive impulses originate from the primary afferents of sensory neurons whose cell bodies are located in the trigeminal and upper cervical ganglia (i.e., first order neurons) [28]. From here, ascending nociceptive information is conveyed to second-order neurons in the brainstem which, in turn, relay the information to third-order neurons in the thalamus [28]. The latter then projects the information to the somatosensory cortex and other cortical and subcortical areas, which are ultimately responsible for the perception of migraine pain and its accompanying symptoms [28].

The initial generation of nociceptive impulses is likely caused by various chemical agents, which are released from primary afferents of the trigeminal and upper cervical ganglia as well as parasympathetic efferents of the sphenopalatine ganglia [2]. These agents are known to promote dilation of meningeal arteries and sensitize perivascular nociceptors [2]. The most well-documented example is calcitonin gene-related peptide (CGRP) [29], albeit it merits emphasis that other agents are also involved.

## Estrogen and the trigeminovascular system

Estrogen is a steroid hormone predominantly synthesized in the ovaries of females [30]. The endogenous natural estrogens include estrone (E1), estradiol (E2), estriol (E3), and estetrol (E4), with estradiol being the primary active estrogen in females of reproductive age [30]. There are three main types of estrogen receptors (ER): ERα, ERβ, and G protein-coupled ER (GPER), all of which are expressed at the level of the trigeminal ganglion and trigeminal nucleus caudalis as well as the hypothalamus [31]. Although it has been suggested that activation of ERs might sensitize trigeminovascular neurons [31], the available data to support this assertion is currently limited.

### The estrogen withdrawal hypothesis

The estrogen withdrawal hypothesis was first described by Sommerville in 1972 [7]. He conducted an interventional study, in which six women with a “regular premenstrual or menstrual migraine” attack received intramuscular (IM) injection of estradiol before the onset of menstruation [7]. All six participants underwent blood sampling before the intervention. Three participants then had daily blood samples collected throughout the menstrual cycle. In the other three participants, daily blood samples were collected during the “*premenstrual and menstrual phases*” (without a clear temporal definition) of two consecutive menstrual cycles. The first cycle served as a control without any intervention, whilst the second cycle was preceded by IM injection of estradiol. In four participants, the intervention was 10-mg estradiol valerate in 3–10 days before the expected onset of menstruation. The remaining two participants received two injections of 5-mg estradiol valerate that were separated by a few days. The results showed that the expected onset of migraine had seemingly been delayed by a few days following the injection of estradiol valerate and, in some, even until after the end of menstrual bleeding. The delayed onset of migraine coincided notably with the precipitous drop in plasma levels of estradiol following a period of sustained high levels as a result of the injection. In addition, the injection increased plasma levels of estradiol but did not affect plasma levels of progesterone nor delay the onset of menstruation. Based on these findings, Sommerville proposed the estrogen withdrawal hypothesis, which states that the decline in plasma levels of estrogen shortly before menstruation increases the risk of developing a migraine attack.

To further explore the possible association of estrogen with menstrual migraine, Sommerville conducted an interventional study [9], in which women who had exclusively premenstrual or menstrual migraine attacks were enrolled. All participants received IM injection of either long-lasting or short-lasting estradiol formulations after the resolution of their usual menstrual migraine attacks. The results showed that three of four women who received long-lasting estradiol developed migraine attacks after plasma levels of estradiol had decreased following a sustained period of high levels. No migraine attacks were reported by the two women who had received short-lasting estradiol. Based on these observations, Sommerville posited that the development of menstrual migraine attacks is associated with decreasing levels of plasma estrogen after a prolonged period of high plasma levels [9]. He therefore asserted that repeated or continuous treatment with estradiol might prevent menstrual migraine attacks [8]. However, he did not find any therapeutic benefits with various formulations of estradiol [8].

**Fig. 2:**
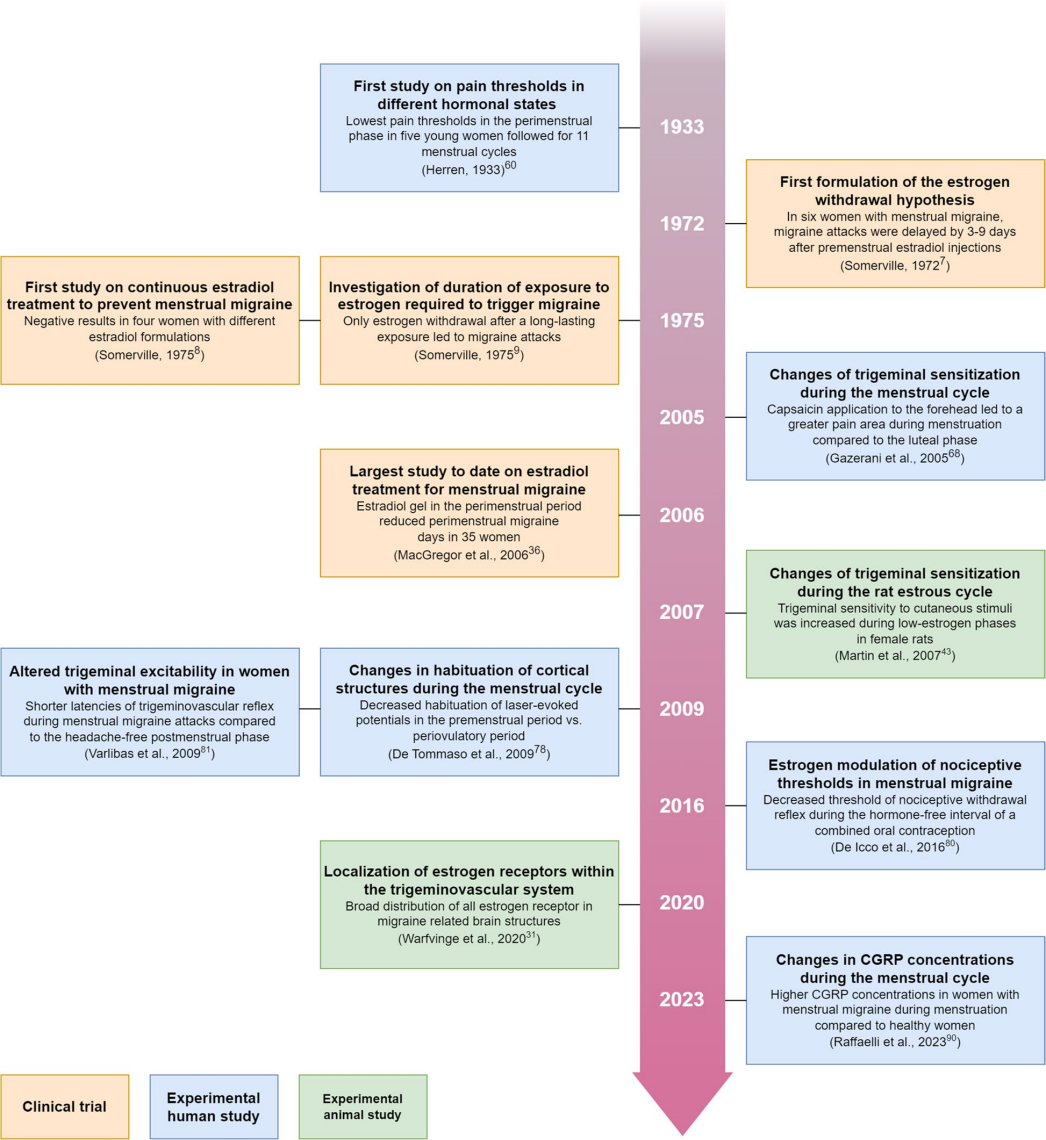
Timeline of key original investigations on the estrogen withdrawal hypothesis in menstrual migraine

The impact of Sommerville’s experiments has been profound, and his estrogen withdrawal hypothesis remains accepted by many in the migraine field (Fig. 2). However, it merits emphasis that his intervention studies were non-randomized, unblinded, and limited by small samples and inconsistent use of case definitions. It seems more reasonable to regard them as idea-generating, exploratory studies that require further validation through more rigorously designed experiments.

### Treating migraine with estradiol

Since the early work by Sommerville [7–9], several clinical trials have been performed to evaluate the effectiveness of treating migraine with estradiol [6]. The administration of perimenstrual estradiol gel in small cohorts of women with menstrual migraine yielded a noteworthy reduction in migraine frequency when compared with placebo [32, 33]. Conversely, two studies that used estradiol patches (50 µg/24 h) reported negative results [34, 35]. The largest study to date was conducted in 2006 and enrolled 35 women with pure menstrual or menstrually-related migraine in adherence with the ICHD-2 criteria [36]. The study had a randomized, placebo-controlled, 2-way crossover design, in which participants were treated with estradiol gel for three menstrual cycles, followed by three cycles of placebo, or vice versa. The treatment period ranged from ten days after the peak fertility, as determined by daily measurements of sex hormones, until the second day of menstruation. Compared to placebo, estradiol treatment was associated with a 22% additional reduction of migraine days during the treatment period. However, the proportion of migraine days during estradiol vs. placebo treatment was only numerically in favor of estradiol but without statistical significance, which might be attributed to the small sample size and the study being underpowered. In line with the original observation by Somerville, migraine frequency increased in the five days after estradiol treatment (RR 1.40 compared to placebo).

The above discussion suggests that the effects of estrogen or its withdrawal on migraine depends on several factors, including dose, duration of exposure, and formulation. However, the available studies are limited by small samples and differences in definitions used. Thus, caution must be exercised in interpreting the current evidence, and further investigation in larger and better-defined samples is needed to ascertain the therapeutic promise of treating migraine with estradiol.

## Insights from experimental studies

Estrogen can modulate several physiological processes in the body, including pain [37]. The effects of estrogen on pain are context-dependent and timing-specific, as it has been shown to possess both analgesic and hyperalgesic properties [37]. The fluctuations in estrogen levels that occur during the menstrual cycle, menopause, or surgical interventions (e.g., ovariectomy) can also affect pain perception [37].

### Insights from animal experiments

Animal studies have produced conflicting findings on the relationship between the estrous cycle and pain perception in rodents [38]. Some studies report reduced pain sensitivity during the proestrus phase when estrogen levels are at their peak [39–42], and that sensitization of the trigeminal nucleus caudalis occurs in the second half of the cycle when estrogen levels are low [43]. Conversely, other animal studies have showed high-estrogen phases to be associated with increased pain sensitivity [44–50].

Ovariectomy is a surgical procedure that involves the removal of ovaries, leading to a significant reduction in estrogen levels. Animal models have demonstrated that ovariectomy can result in a decrease in pain threshold for various pain stimuli [51–53]. This effect can then be reversed by estrogen supplementation [51, 53–56]. However, there are also studies reporting no change or an increase in pain perception following ovariectomy in animal models [57, 58]. Therefore, the direction in which estrogen modulates pain perception remains unclear [37]. Some discrepancies might be explained by a site-specific hormonal sensitivity in nociception, with low estrogen states causing increased orofacial pain sensitivity with less effects on extracephalic pain [59].

It is uncertain whether findings from animal studies can be directly applied to migraine pain in humans [37]. This uncertainty stems from several factors. Firstly, the hormonal profile of the rodent estrous cycle differs from that of the human menstrual cycle, which limits comparisons [37]. Secondly, most animal studies have examined pain sensitivity in non-cephalic areas of the body [37], and thus may not reflect the perception of head pain characteristic of migraine. Lastly, migraine is a unique human experience, underscoring the importance of research involving humans to draw conclusive results.

### Insights from human experiments

The menstrual cycle’s influence on pain perception was first reported by Herren in 1933 [60]. He studied five young women over 11 menstrual cycles and discovered that pain and touch two-point discrimination thresholds were lowest during the premenstrual phase. Later studies validated these findings by demonstrating that pain sensitivity to different kind of stimuli is higher during low estrogen phases and lower during high estrogen phases [61–67]. In one study, capsaicin was injected on the forehead of 14 healthy females during both their menstrual and luteal phases [68]. The pain area, as drawn by participants on a face chart, as well as the area of facial redness mapped by the investigators were larger during menstruation, which might indicate increased trigeminal sensitization during this phase. However, some studies have reported pain threshold changes in the opposite direction [69, 70], while others found no cyclical changes of pain thresholds in women [71–77].

Another line of experiments evaluated the laser-evoked potential (LEP) in the premenstrual period (i.e., 1–2 days prior to menstruation) and periovulatory period (i.e., 13–15 days prior to menstruation) of nine women with migraine and ten healthy controls [78]. Trigeminal nociception was tested by stimulating the supraorbital zone, while non-trigeminal nociception was assessed by stimulating the right hand. The results showed that women with migraine had an increased amplitude and reduced habituation of LEP in both phases, compared with healthy controls at both sites of testing. In addition, both groups had an increased amplitude and decreased habituation of LEP in the premenstrual phase. The authors suggested that reduced habituation of pain-relevant cortical structures prior to menstruation might increase the likelihood of developing migraine attacks. However, a study involving 32 women with migraine and 20 healthy controls did not find any differences in conditioned pain modulation across the menstrual cycle [79].

Two studies have provided insight into the possible association of nociceptive changes with menstrual migraine. In one study, 11 women were enrolled and all experienced migraine attacks exclusively during the hormone-free period when on a combined oral contraceptive regimen. This regimen entailed 21 days of hormone intake, followed by a 7-day hormone-free period (i.e., 21/7 regimen) [80]. The authors measured the nociceptive withdrawal reflex after sural nerve stimulation during the third week of active treatment and during the estrogen withdrawal period. A decrease in the reflex threshold was observed during the hormone-free interval, which underscores the potential roles of estrogen in modulating pain perception in menstrual migraine. In the other study involving 31 patients with pure menstrual or menstrually-related migraine, the trigeminovascular reflex was evaluated during menstruation and in the headache-free postmenstrual phase [81]. The authors observed shorter reflex latencies during menstruation, indicating a hormonal modulation in brainstem excitability. However, these changes might reflect differences between ictal and interictal phase of migraine rather than being related to hormonal factors, and further investigation is thus needed.

The human experimental studies into estrogen-dependent pain sensitivity remain inconclusive, likely due to differences in characteristics of the study populations and methodology. Despite this, some evidence does indicate that estrogen withdrawal facilitates pro-nociceptive responses, thereby potentially increasing the susceptibility to develop migraine attacks. To arrive at conclusive results, further research with larger sample sizes and standardized methodology is required.

### Calcitonin gene-related peptide

The available evidence indicate that estrogen might modulate nociception in migraine, possibly by affecting chemical agents implicated in the genesis of cephalic pain. Among these, CGRP is the most extensively studied agent of interest [82]. The role of CGRP within the trigeminovascular system has been extensively investigated for almost four decades [29]. CGRP is released from primary afferents in the trigeminovascular system [83], and intravenous infusion of CGRP induces migraine attacks in people with migraine [84]. Furthermore, blocking CGRP signaling has proven effective for the acute and preventive treatment of migraine [29].

In some in vitro animal studies, estradiol has been found to reduce CGRP expression in the trigeminal ganglion and trigeminal nucleus caudalis of rodents, whereas ovariectomy led to increased CGRP gene expression [82]. It does however merit mention that other animal studies failed to detect any effect of estrogen on the trigeminovascular system [82].

The relationship between estrogen and CGRP in humans remains unclear, as studies have produced conflicting evidence. One small study reported higher CGRP plasma immunoreactivity in 24 females than in 13 males, with women using combined contraception having the highest CGRP concentrations [85]. However, the study did not account for menstrual cycle phase or hormonal levels. Another study with 55 women in different pregnancy states reported higher estrogen concentrations during pregnancy that decreased postpartum [86]. In healthy women, application of capsaicin to the forehead led to a higher increase in CGRP-dependent dermal blood flow measured by laser doppler perfusion imaging during menstruation compared to ovulation or other times of the menstrual cycle [87]. Other studies have, however, suggested the opposite relationship between estrogen and CGRP [88, 89]. Of note, a recent study reported higher interictal CGRP concentrations in the plasma and tear fluid of women with menstrually-related migraine during menstruation in comparison to healthy women, which could explain their heightened susceptibility to migraine during the perimenstrual period [90]. Further studies are, nonetheless, required to validate these findings. Interpretation of CGRP findings in humans must account for the potential for discrepancies and uncertainties between studies, which can arise due to differences in populations, biomaterials, and methodology. Overall, the current evidence suggests that a dysfunction in the modulation of the CGRP pathway might play a role in the development of menstrual migraine, albeit this assertion is based on preliminary findings. The intricate relationship between CGRP and estrogen offers a compelling direction for future research. It seems pertinent to determine if estrogen can modulate the responsiveness of meningeal nociceptors in rodents, both with and without the administration of CGRP. In addition to this, human experimental studies might evaluate if pre-treatment with estrogen can reduce the incidence of migraine attacks after intravenous infusion of CGRP. Such insights might advance our understanding of migraine pathogenesis and, in particular, the origins of perimenstrual attacks.

### Genetic insights

The role of genetics in migraine is an active area of research [91], with some studies suggesting that genetic factors might be involved in the neurobiologic underpinnings of menstrual migraine [92]. Genetics might indeed regulate individual-level sensitivity to estrogen fluctuations, making some women more susceptible to develop menstrual migraine [92]. The current evidence is however conflicting, with, one meta-analysis finding two estrogen receptor polymorphisms (ESR-1 594 G > A and ESR-1 325 C > G) linked to migraine [93], whilst a recent genome-wide association study did not identify any specific hormonal factors among 123 loci connected to migraine [94]. These data derive from pooled populations of patients with all subtypes of migraine. Therefore, conclusive remarks cannot be made for those individuals with menstrual migraine based on these observations.

Limited evidence exists for the role of genetics in menstrual migraine which, in part, is attributed to the available data coming from small cohorts that use different case definitions. A British study reported no significant difference in functional polymorphisms of estrogen synthesis and metabolism genes (COMT, CYP1A1, and CYP19A1) between 268 women with menstrual migraine and 142 healthy controls [95]. This finding aligns well with an Italian study finding no association between COMT polymorphisms and pure menstrual or menstrually-related migraine in a cohort of 380 participants with migraine and 132 healthy individuals [96]. However, an American study identified one COMT polymorphism (rs4680) and two tyrosine hydroxylase gene polymorphisms (TH rs2070762 and TH rs6356) linked to self-reported menstrual migraine [97]. Moreover, two ESR-1 polymorphisms (rs2234693 and rs726281) were associated with menstrually-related migraine in Chinese and Turkish cohorts [98, 99]. A British analysis of 37 genetic variants in 14 genes also showed a significant association between menstrual migraine and genetic polymorphisms in the TNF and SYNE1 genes, while NRP1 is another potentially involved gene, with one polymorphism linked to menstrual migraine in the same British cohort [100]. It should be noted that each identified variant is likely to account for modest effects in increasing the risk of developing menstrual migraine. More research is therefore warranted to fully ascertain the role of genetics in menstrual migraine.

## Conclusions

The estrogen-withdrawal hypothesis has been the leading theory to explain menstrual migraine since it was first proposed in the 1970s. However, a critical reappraisal reveals limited and conflicting evidence to support this hypothesis based on the available animal and human experimental studies. While it seems evident that estrogen fluctuations are linked to migraine pathophysiology, the exact mechanisms are still a subject to debate. Future research should focus on elucidating the pathogenesis of menstrual migraine in larger and well-defined human cohorts using consistent methodological procedures.

## Data Availability

Not applicable.

## Abbreviations

CGRP: Calcitonin Gene-Related Peptide
ER: Estrogen receptor
GPER: G protein-coupled estrogen receptor
ICHD: International Classification of Headache Disorders
IM: Intramuscular
LEP: Laser-evoked potential
